# Innate Immune Signaling by, and Genetic Adjuvants for DNA Vaccination

**DOI:** 10.3390/vaccines1030278

**Published:** 2013-07-18

**Authors:** Kouji Kobiyama, Nao Jounai, Taiki Aoshi, Miyuki Tozuka, Fumihiko Takeshita, Cevayir Coban, Ken J. Ishii

**Affiliations:** 1 Laboratory of Adjuvant Innovation, National Institute of Biomedical Innovation, 7-6-8 Saito-asagi, Ibaraki, Osaka 567-0085, Japan; E-Mails: kobi@nibio.go.jp (K.K.); n-jonai@nibio.go.jp (N.J.); t-aoshi@nibio.go.jp (T.A.); tozuka-miyuki@nibio.go.jp (M.T.); takeshita.fumihiko@japanvaccine.co.jp (F.T.); 2 Laboratory of Vaccine Science, Immunology Frontier Research Center, Osaka University, 3-1 Yamadaoka, Suita, Osaka 567-0871, Japan; 3 Laboratory of Malaria Immunology, Immunology Frontier Research Center, Osaka University, 3-1 Yamadaoka, Suita, Osaka 567-0871, Japan; E-Mail: ccoban@biken.osaka-u.ac.jp

**Keywords:** DNA vaccine, innate immune responses, adjuvant, DNA sensor

## Abstract

DNA vaccines can induce both humoral and cellular immune responses. Although some DNA vaccines are already licensed for infectious diseases in animals, they are not licensed for human use because the risk and benefit of DNA vaccines is still controversial. Indeed, in humans, the immunogenicity of DNA vaccines is lower than that of other traditional vaccines. To develop the use of DNA vaccines in the clinic, various approaches are in progress to enhance or improve the immunogenicity of DNA vaccines. Recent studies have shown that immunogenicity of DNA vaccines are regulated by innate immune responses via plasmid DNA recognition through the STING-TBK1 signaling cascade. Similarly, molecules that act as dsDNA sensors that activate innate immune responses through STING-TBK1 have been identified and used as genetic adjuvants to enhance DNA vaccine immunogenicity in mouse models. However, the mechanisms that induce innate immune responses by DNA vaccines are still unclear. In this review, we will discuss innate immune signaling upon DNA vaccination and genetic adjuvants of innate immune signaling molecules.

## 1. Introduction of DNA Vaccines

Almost two decades ago, it was reported that plasmid DNA could induce adaptive immune responses against plasmid-encoded antigens [1], indicating it could be used in novel therapeutic applications as a human vaccine for the prevention of various pathogen infections [2], autoimmunity [3], allergy [4], neurological disorders [5], and cancer [6]. In the veterinary field, some DNA vaccines are already licensed for West Nile virus in horse, infectious hematopoietic necrosis virus in salmon, and melanoma in dogs [7]. For human use, DNA vaccines have not been licensed, however, many candidate DNA vaccines are being studied in ongoing clinical trials. The clinical benefits of DNA vaccine are low cost, vaccine stability, high productivity, and easy modification of antigen in comparison with traditional protein vaccines. Conversely, it was reported that the immunogenicity of DNA vaccines was quite low according in clinical trials. Indeed, the immunogenicity of DNA vaccines tended to be weaker than other types of vaccines using live virus, virus vectors, or traditional protein plus adjuvant vaccines. Therefore, the immunogenicity of DNA vaccines was improved by changing promoters, codon usage of antigen sequences, the insertion of genetic adjuvants such as cytokines and innate immune activation molecules, strategies to prime and boost vaccination, and the route of administration [8].

Furthermore, elucidation of the molecular mechanisms of DNA vaccines is also important for developing DNA vaccines for human use. TANK-binding kinase 1 (TBK1), and stimulator of interferon genes (STING), was identified as an essential molecule for the induction of adaptive immune responses by DNA vaccination. In addition, double-stranded DNA (dsDNA) is a critical ligand of the STING-TBK1 signaling cascade [9]. These results indicate that dsDNA-induced innate immune signaling lead to induction of DNA-encoded antigen specific adaptive immune responses, like an adjuvant. However, DNA sensing machinery is still controversial. In this review, we will discuss innate immune signaling of DNA vaccines and genetic adjuvants of innate immune signaling molecules.

In 1990, Wolf *et al*. showed that the intramuscular administration of naked DNA led to the induction of DNA-encoded reporter genes in muscle cells [10]. Subsequently, Ulmer *et al*. demonstrated that the intramuscular administration of plasmid DNA encoding influenza viral protein induced encoded antigen-specific cytotoxic T lymphocyte (CTL) responses, which protected against lethal influenza virus infection [1]. These findings were the first evidence that naked DNA administration alone could induce adaptive immune responses against antigens expressed from plasmid DNA, and suggested that DNA vaccine strategies might be useful for clinical use. Indeed, many researchers evaluated novel DNA vaccines using experimental infectious diseases models [11]. The properties of DNA vaccines represent greater stability, low cost, high productivity, and possibility to improve immunogenicity. In 1998, the first human clinical trial of DNA vaccines against human immunodeficiency virus was reported [12]. This study evaluated the safety and efficacy of DNA vaccines. Importantly, one of the safety concerns for DNA vaccines was the integration of plasmid DNA into the host genome [13]. If integration occurs following DNA vaccination, the integrated-DNA may cause oncogene activation, tumor suppressor gene inactivation, or chromosomal instability. Fortunately, experimental data showed the rate of plasmid DNA integration was lower than the natural rate of mutation in mammalian genomes [14]. Another safety concern is development of anti-DNA antibodies, associated with autoimmune disorders [15]. Anti-dsDNA antibody was increased in mouse after DNA vaccination [16]. In the clinical trials, anti-DNA antibody did not increase in any study subject [17]. However, the improvement of DNA vaccines to enhance immunogenicity may increase the risk of integration and development of anti-DNA antibody. Therefore, evaluation of safety concerns is essential before clinical trials are initiated. Subsequently, research groups have developed novel DNA vaccines against cancer, influenza virus, human papillomavirus, hepatitis, and malaria. However, the early clinical trials showed disappointing results. 

### 1.1. Mode of Action

Although DNA vaccines can induce both humoral and cellular immune responses against plasmid-encoded antigens, the mode of action of DNA vaccines is still unclear. However, when DNA plasmids are administered to muscle, skin, subcutaneous, or the nasal cavity, it is believed that the DNA plasmid enters cells, translocates to the nucleus, and antigen is expressed by the host cellular machinery. In most cases, myocytes and antigen presenting cells (APCs), such as dendritic cells (DCs) or macrophages, appear to capture plasmid DNA. Subsequently, antigen protein is degraded and presented by major histocompatibility complex (MHC)-I in immune cells. Additionally, expressed-antigens can be secreted from cells by active secretion of the protein or released due to apoptosis of the transfected cell. Secreted antigen proteins are taken up, degraded, and presented by APCs on MHC-I and MHC-II molecules. Finally, APCs recruited to the draining lymph nodes activate naïve B cells, CD4+ and CD8+ T cells. In many cases, secreted antigen proteins could induce both IgG1 and IgG2a/c antibody, and cytosolic protein antigens could induce IgG2a/c antibody.

### 1.2. Methods of DNA Vaccination

Intramuscular electroporation (imEPT) is one method of DNA vaccine administration, which overcomes limitations such as low transfection efficacy and insufficient recruitment of APCs to the injection site, by inducing transient enhancement of cell membrane permeability. Consequently, the increased uptake of DNA into the host cell and induction of low level of inflammation can enhance the influx of APCs to the injection site [18]. This method induces potent immune responses including CTL responses, and is therefore a convenient method for analyzing the intracellular signaling cascade of DNA vaccines. Indeed, for most cases, the contribution of innate immune activation by DNA vaccination is evaluated using imEPT in mouse models. Gene gun [19], needle-free systems [20], and mucosal delivery [21] are studied as other methods for DNA vaccination; however, these methods have not been examined to elucidate the innate immune signaling of DNA vaccination. It is important whether these vaccination methods activate same innate immune signaling cascade. 

## 2. Innate Immunity and DNA Vaccines

### 2.1. Immunostimulatory Properties of Double-Stranded DNA

At present, it is known that nucleic acids such as DNA and RNA induce innate immune responses such as type I interferon (IFN) and inflammatory cytokine production. Interestingly, the innate immune activation of DNA is affected by DNA structure and conformation. In 1963, it was reported that rat liver derived-DNA or RNA stimulation could produce type I IFN from chick cells [22]. In 1984, Bacillus Calmette-Guérin-derived DNA was shown to have strong anti-tumor activity [23]. These findings were the first evidence that both host and bacterial DNA induced innate and adaptive immune responses. Subsequently, bacteria-derived unmethylated CpG DNA and synthetic CpG oligonucleotide (ODN) were shown to be direct stimulators of B cells [24]. Additionally, Toll-like receptor 9 (TLR9) was identified as a receptor for CpG motif DNA that activated innate immune responses in immune cells, such as DCs, B cells, and macrophages [25]. Meanwhile, host DNA-induced innate immune activation was forgotten and ignored. In 1999, the independent effects of unmethylated CpG motifs or specific DNA sequences were shown as at least 25 base pairs of synthetic double-stranded (ds), but not single-stranded (ss) DNA up-regulated the expression of genes related to immune responses [26]. Later, the B-form conformation of dsDNA was shown to be more effective at inducing innate immune responses than the Z-form of dsDNA [27]. Stimulation with synthetic B-form dsDNA, poly (dA-dT) poly (dA-dT), resulted in the induction of type I IFN and IFN-inducible chemokines, whereas stimulation with synthetic Z-form dsDNA, brominated poly (dG-dC) poly (dG-dC) only induced CXCL10 release. 

Studies then focused on adaptive immune responses and demonstrated genomic DNA derived from dead cells induced the maturation of APCs and cellular immune responses, especially CTL responses [28]. In addition, traditional aluminum adjuvant induced cell death and host-derived DNA release, which induced antigen specific IgE production [29]. These results indicate that the immunostimulatory effect of self-DNA could cause the induction of innate immune responses and side-effects in the host. Adverse effects of aberrant DNA have been shown in relation to the function of DNase, an enzyme that digests DNA. DNase II-deficient mice failed to digest DNA from engulfed nuclei of erythroblasts in hepatic macrophages and resulted in the robust production of type I IFN and inflammatory cytokines, which caused severe anemia and rheumatoid arthritis (RA)-like symptoms in a TLR9-independent manner [30,31]. DNase I and DNase III knockout mice developed systemic lupus erythematosis-like symptoms and inflammatory myocarditis, respectively [32,33,34]. The functional mutations of DNase I and DNase III in humans were also shown to cause several autoimmune disorders, such as systemic lupus erythematosis [33,35], Aicardi-Goutieres syndrome [36], familial chilblain lupus [37], or retinal vasculopathy with cerebral leukodystrophy [38]. Thus, DNA-induced immune responses are not only involved in the prevention of microbial infection but also of autoimmune responses. These findings indicate that normal cells are equipped with innate sensing machineries to remove aberrant genomic DNA fragments.

### 2.2. Cellular Signaling of DNA Vaccines

In general, DNA vaccines derived from bacterial plasmids contain unmethylated CpG motifs recognized by TLR9, which induce innate immune responses [25]. Therefore, many researchers have attempted to clarify whether TLR9-induced innate immune responses are required for immunogenicity of DNA vaccines. Unexpectedly, some reports suggested that TLR9 was not essential for the induction of immune responses of DNA vaccines *in vivo*, although plasmid-induced cytokine production from immune cells was completely dependent on TLR9 *in vitro* [39,40]. Importantly, dsDNA, including plasmid DNA, could activate both immune cells and non-immune cells such as fibroblasts or keratinocytes. Therefore, TLR9-independent DNA sensing machinery might also be involved in the immunogenicity of DNA vaccines [39,40]. 

TBK1 is noncanonical IκB kinase that directly phosphorylates interferon regulatory factor 3 (IRF3) to produce type I IFN by TLR-dependent and -independent pathways [27,41]. Thus, TBK1 is important for the activation of innate immune responses upon pathogen infection, tumor development, or autoimmune disease. TBK1-deficient mouse embryonic fibroblasts (MEFs) do not induce cytokine production when stimulated with B-form DNA [27]. Interestingly, TBK1-deficient mice were not able to induce either humoral or cellular immune responses upon DNA vaccination [42]. In addition, type I IFN receptor-deficient mice also showed abolished induction of adaptive immune responses. These results strongly suggest that TBK1-dependent but TLR9-independent mechanisms for the type I IFN signaling cascade are critical for the induction of adaptive immune responses following DNA vaccination. Another important molecule is STING (also known as MITA, ERIS, and MYPS) [43,44,45,46] that was firstly reported to be associated with MHC-II-mediated cell death [37]. Subsequently, STING was shown to function as an adaptor molecule that activates innate immune signaling upon cytosolic dsDNA recognition [43]. STING-deficient MEFs did not activate dsDNA-mediated innate immune signaling. Furthermore, STING deficient mice could not induce humoral and cellular immune responses by DNA vaccination [47]. Surprisingly, a recent study showed that STING directly binds to dsDNA to induce innate immune activation [48]. However, it is still unclear whether STING directly binds to plasmid DNA and contributes to DNA vaccine immunogenicity. Other innate immune signaling molecules have been evaluated for their involvement in DNA vaccine immunogenicity and demonstrated that IRF3 is only involved in cellular immune responses but not humoral immune responses [49]. Although STING and TBK1 studies were examined by imEPT to evaluate their contribution to the immunogenicity of the DNA vaccine, IRF3 research has not used the electroporation method. Studies indicate that dsDNA-mediated, but not TLR9-dependent, innate immune signaling regulates the immunogenicity of DNA vaccines [42,47]. Interestingly, our preliminary data showed that other transcription factors are involved in the immunogenicity of DNA vaccines, which are dependent on antigen properties [50].

### 2.3. Cytosolic Sensors for DNA Fragments and Their Metabolites

**Figure 1:**
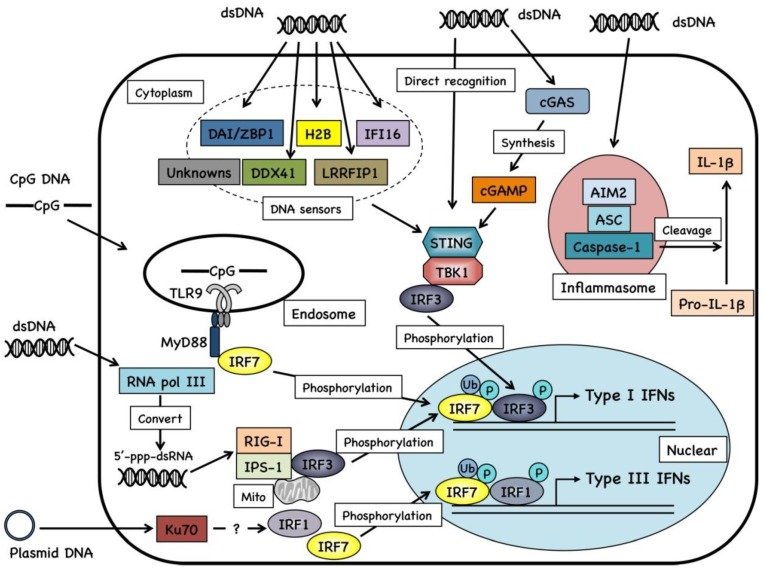
Cytosolic DNA sensing machinery.

To date, several cellular molecules are reported as DNA sensors that recognize aberrant cytosolic DNA (Figure 1). These sensors are involved in the elimination of invasive pathogens, and induce innate immune signaling. In most cases, recognition of cytosolic DNA by these sensors results in the induction of innate immune responses through the STING-TBK1 signaling cascade [27,43], suggesting that the detection of dsDNA structure of plasmid DNA by cytosolic DNA sensing machinery contributes to the enhanced adaptive immune responses against DNA vaccine-encoded antigens.

Z-DNA binding protein 1/DNA-dependent activator of IFN-regulatory factors (ZBP1/DAI) was reported as the first cytosolic dsDNA sensor [51]. Overexpression of ZBP1/DAI increased type I IFN gene expression by dsDNA stimulation such as bacterial and mammalian DNA. Knockdown of ZBP1/DAI resulted in decreased IFN-β production by dsDNA and DNA virus infection but not synthetic dsRNA and RNA virus infection. In addition, ZBP1/DAI directly interacted with B-form DNA in the cytoplasm. Of interest, however, ZBP1/DAI deficient MEFs responded normally to dsDNA, and ZBP1/DAI deficient mice showed normal adaptive immune responses against DNA-encoded antigen [42].

Retinoic acid-inducible gene I (RIG-I), and melanoma differentiation-associated gene 5 (MDA5) were identified as cytosolic RNA sensors and activated innate immune responses to protect RNA virus infection [52]. These receptor-mediated signaling pathways are completely regulated by adaptor molecule IFN-β promoter stimulator 1 (IPS-1) (also known as MAVS, VISA, and Cardif) [53,54,55,56]. Although RIG-I acts as a cytosolic RNA receptor, it was shown to be involved in the indirect recognition of cytosolic dsDNA. Knockdown of RIG-I resulted in reduced type I IFN production by both dsDNA and dsRNA stimulation in a human hepatocellular carcinoma cell line, HuH-7. Subsequently, it was shown that RNA polymerase III transcribed 5'-triphosphate RNA from poly(dA·dT)·poly(dT·dA) or pathogen genome DNAs as a template, and facilitated the RIG-I-mediated type I IFN production cascade. Intracellular bacteria-induced type I IFN production was abrogated by inhibitors of specific RNA polymerase III, resulting in the promotion of bacterial growth [57]. Although RIG-I-mediated innate immune signaling is completely regulated by IPS-1, IPS-1-deficient mice had normal adaptive immune responses against plasmid DNA vaccinations [42]. In addition, at least in human cells, knockdown of IPS-1 resulted in decreased type I IFN production after dsDNA stimulation [27]. The involvement of RIG-I-IPS-1 signaling in human DNA vaccination is still controversial.

Double stranded DNA induces both innate immune responses and cell death. It was reported that electroporated DNA could induce cell death in murine macrophages [58]. Absence in melanoma 2 (AIM2) was identified as a cytosolic DNA sensor that activated the inflammasome to produce IL-1β and dsDNA-induced cell death. On recognition of cytosolic dsDNA, AIM2 interacts with inflammasome-related molecules to induce pyroptosis, a type of programmed cell death characterized by the activation of caspase-1 and IL-1β production. Deficiency of AIM2 resulted in enhanced susceptibility to bacteria and DNA virus [59,60]. Collectively, electroporation of plasmid DNA might cause aberrant DNA to induce inflammasome activation or cytokine production via AIM2. 

Histone H2B is a component of chromatin. Recently, we demonstrated that histone H2B recognized dsDNA in the cytosol to induce innate immune responses through IPS-1 and COOH-terminal importin 9-related adaptor organizing histone H2B and IPS-1 (CIAO). In addition, histone H2B sensed host-derived dsDNA after cell damage by electroporation [61]. Taken together, histone H2B might contribute to the recognition of administered plasmid DNA and electroporated-derived DNA to induce adaptive immune responses against DNA vaccines. In addition, interferon gamma inducible protein 16 (IFI16) [62], high mobility group box protein 1 (HMGB1) [63], Ku70 [64], leucine-rich repeat flightless-interacting protein 1 (LRRFIP1) [65], and DDX41 [66] were also identified as cytosolic DNA sensors. 

Nucleotide second messenger, cyclic-di-GMP, is synthesized by bacteria from two GTP precursors and induced innate immune activation through the STING-TBK1 signaling cascade [67]. Recently, it was reported that after DNA transfection or DNA virus infection cyclic GMP-AMP (cGAMP) was produced by cGAMP synthase (cGAS), a member of the nucleotidyltransferase family. This endogenous nucleotide second messenger induced innate immune responses. Indeed, cGAS binds to DNA in the cytoplasm and catalyzes cGAMP synthesis to act as a cytosolic dsDNA sensor [68]. Furthermore, cGAMP directly interacted with STING to activate IRF3, and knockdown of cGAS suppressed IFN-β production by dsDNA transfection or DNA virus infection. It will be interesting to examine whether DNA vaccination induces cGAMP using plasmid DNA as a template to induce adaptive immune responses.

Studies of DNA sensors were performed using different cell types, synthetic DNAs, bacteria, and viruses. However, only limited type of knockout mice have been used for DNA vaccines, although DNA-mediated innate immune signaling is related to the immunogenicity of DNA vaccines. To elucidate which DNA sensors contribute to the immunogenicity of DNA vaccines, the data by using various DNA sensor gene-deficient mice should be accumulated.

## 3. Genetic Adjuvant

### Innate Immune Activation Molecules

In general, the immunogenicity of DNA vaccines is lower than for traditional protein vaccines or live vaccines, although DNA vaccines contain a “built-in” adjuvant, the CpG motif. Indeed, addition of several CpG motifs into plasmid DNA resulted in improved immunogenicity of DNA vaccines [69]. Additionally, human specific CpG motifs containing DNA vaccines induced the maturation of human monocytes [70] suggesting that improvements to plasmid DNA for innate immune signaling activation are important for the enhancement of immunogenicity and induction of optimal immune responses. 

Recently, TLR adaptor molecules, such as myeloid differentiation primary response gene (MyD88) and Toll/IL-1 receptor (TIR)-domain-containing adaptor inducing interferon-β (TRIF) was inserted into plasmid DNA as a genetic adjuvant and enhanced humoral immune responses against plasmid-encoded antigen (Table 1). In contrast, TRIF genetic adjuvant potently enhanced cellular immune responses. Indeed, TRIF genetic adjuvant elicited protection against lethal influenza virus infection and tumor progression [71]. These studies suggest that TLR agonists may act as DNA vaccine adjuvants.

Flagellin is a TLR5 agonist that activates innate immune responses. Dermal injection of plasmids encoding flagellin, and influenza A virus nucleoprotein enhanced both humoral and cellular immune responses. Interestingly, the flagellin vaccine adjuvant induced antigen-specific IgA production and enhanced protective immunity to lethal influenza A virus infection [72]. These results demonstrate that expression of DNA-encoded TLR agonists can improve the immunogenicity of DNA vaccines.

In addition, IRF1, 3, and 7 were also evaluated as genetic adjuvants for influenza virus DNA vaccines. IRF1 genetic adjuvant strongly enhanced humoral immune responses. In contrast, IRF3 genetic adjuvant induced stronger cellular immune responses. Interestingly, IRF7 genetic adjuvant enhanced both humoral and cellular immune responses [73]. These results suggest that IRF genetic adjuvants can improve both humoral and/or cellular immune responses. In addition, constitutive active forms of IRF3 and IRF7 were evaluated as DNA vaccine adjuvants and elicited both humoral and cellular immune responses to protect against vaccinia virus infection [74]. Furthermore, DNA binding domain-lacked IRF1 (ΔIRF1) was superior to full length IRF1 on HIV TAT DNA vaccines, as ΔIRF1 genetic adjuvant enhanced cellular immune responses [75]. 

Recently, we showed that TBK1 acts as a genetic DNA vaccine adjuvant. *Plasmodium falciparum* serine repeat antigen 36 (SERA36)-encoded DNA vaccine administration with TBK1 genetic adjuvant enhanced at least humoral immune responses but not detect any cellular immune responses in this immunization [76]. These results suggest that TBK1 genetic adjuvant improves the immunogenicity of DNA vaccines, at least in anti-malarial immunogenicity. 

It was reported that ZBP1/DAI interacted with receptor-interacting protein kinase 3 to mediate virus-induced necrosis [77], and electroporated DAI-encoded plasmid DNA facilitated the transcription of type I IFN and proinflammatory cytokines *in vivo*. In addition, DAI genetic adjuvant enhanced CTL responses by type I IFN and NF-κB-dependent but IRF3-independent mechanisms. Co-administration of DAI-encoded plasmid with melanoma-associated antigen tyrosinase-related protein-2 (TRP2) DNA vaccine resulted in enhanced tumor rejection and protection against B16 melanoma challenge [78]. However, whether the improvement of DNA vaccine immunogenicity involves DAI-mediated cell death is still unclear. These results suggest that at least DAI genetic adjuvant can improve the immunogenicity of DNA vaccines.

HMGB1 was also evaluated as a genetic adjuvant for DNA vaccines. Co-immunization with HMGB1 expressing plasmid with HIV-1 Gag and Env expressing DNA vaccines resulted in enhanced humoral and cellular immune responses [79]. In addition, HMGB1 genetic adjuvant also enhanced the immunogenicity of influenza DNA vaccines [80]. Furthermore, chicken (chMDA5) acted as a genetic adjuvant for avian H5N1 influenza virus DNA vaccine. MDA 5 is a RIG-I like receptor that recognizes cytosolic RNAs to induce innate immune responses. In chickens, MDA5 seems to recognize avian influenza virus infection, because chickens lack RIG-I. chMDA5 genetic adjuvant enhanced humoral immune responses and protected against a lethal H5N1 infection [81]. 

**Table 1:** Adjuvant effects of innate immune signaling molecules.

Genetic Adjuvant	DNA vaccine-induced immune responses			Vaccine model	Reference
	Ab* responses	CD4+ T cells	CD8+ T cells		
MyD88	↑↑	Not tested	↑	Tumor, Influenza	[71]
TRIF	↑	Not tested	↑↑	Tumor, Influenza	[71]
IRF1	↑↑	↑	↑	Influenza	[73]
ΔIRF1	→	↑	↑↑	HIV-1	[75]
IRF3	↑	↑↑	↑↑	Influenza	[73,74]
IRF7	↑	↑	↑	Influenza	[73,74]
Flagellin	↑	↑	↑↑	Influenza	[72]
TBK1	↑	→	→	Malaria	[76]
HMGB1	↑	↑	↑	HIV, Influenza	[79,80]
DAI/ZBP1	Not tested	Not tested	↑	Tumor	[78]
chMDA5	↑	Not tested	Not tested	Avian Influenza	[81]

*Ab, antibody.

## 4. Conclusions

About 15 years have passed since the first human clinical trial for DNA vaccines. At present, DNA vaccines are not yet approved for human use. However, many researchers have attempted to improve plasmid DNA, using codon optimization, proper antigen selection, localization changes and addition of antigen signal sequences, appropriate delivery systems and routes, cytokines, and costimulatory molecules as adjuvants, innate immune signaling molecules as adjuvants, targeting for vaccine delivery systems and presentation, and prime boost strategies, amongst others. Indeed, some approaches have succeeded in improving the immunogenicity of DNA vaccines. However, it is important to elucidate the modes of action, such as the cellular and intracellular mechanisms of DNA vaccines. Currently, only dsDNA-mediated STING/TBK1 signaling cascade has been shown to mediate the induction of adaptive immune responses by DNA vaccination. Therefore, it is important to understand how to recognize and induce innate and adaptive immune responses to develop novel, safe, and effective DNA vaccines. 
